# Schisandrin A restrains osteoclastogenesis by inhibiting reactive oxygen species and activating Nrf2 signalling

**DOI:** 10.1111/cpr.12882

**Published:** 2020-09-01

**Authors:** Shuo Ni, Zhi Qian, Yin Yuan, Dejian Li, Zeyuan Zhong, Farnaz Ghorbani, Xu Zhang, Fangxue Zhang, Zhenhua Zhang, Zichen Liu, Baoqing Yu

**Affiliations:** ^1^ Department of Orthopedics Shanghai Pudong Hospital Fudan University Pudong Medical Center Shanghai China; ^2^ State Key Laboratory for Diagnosis and Treatment of Infectious Diseases The First Affiliated Hospital School of Medicine Zhejiang University Hangzhou China; ^3^ School of Materials Science and Engineering University of Shanghai for Science and Technology Shanghai China

**Keywords:** Nrf2, osteoclastogenesis, ROS, schisandrin A

## Abstract

**Objectives:**

Intracellular reactive oxygen species (ROS) induced by receptor activator of NF‐kB ligand (RANKL) has been proven to be a critical factor in the development of osteoclasts. This study aimed to prove that schisandrin A (Sch), a novel anti‐oxidant compound, is able to suppress osteoclastogenesis and prevent bone loss in ovariectomized (OVX) mice by suppressing ROS via nuclear factor erythroid 2‐related factor (Nrf2).

**Material and Methods:**

Micro‐CT was used to detect bone formation. The effects of Sch on receptor activator of nuclear factor‐κB (NF‐κB) ligand (RANKL)‐induced reactive oxygen species (ROS) were measured by dihydroethidium (DHE) staining in vivo and 2',7'‐dichlorodihydrofluorescein diacetate (DCFH‐DA) staining in vitro. Immunofluorescence staining was used to detect the expression of Nrf2 in vivo. siRNA was used to evaluate the effect of Nrf2 in osteoclastogenesis.

**Results:**

Sch suppresses RANKL‐induced ROS production by regulating nuclear factor erythroid 2‐related factor (Nrf2) in vitro and vivo. Mechanistically, Sch enhances the expression of Nrf2 by regulating the degradation of Nrf2. Further, Sch suppresses phosphorylation of P65 and its nuclear translocation, as well as the degradation of IκBα. Collectively, our findings reveal that Sch protects against OVX‐induced bone loss by suppressing ROS via Nrf2.

**Conclusions:**

Our results showed the potential of anti‐oxidant compound schisandrin A in the treatment of osteoporosis, highlighting Nrf2 as a novel promising target in osteoclast‐related disease.

## Graphical abstract

Proposed scheme of Sch restrains osteoclastogenesis by inhibiting reactive oxygen species and activating Nrf2 signalling. Sch suppresses RANKL‐induced ROS production by regulating nuclear factor erythroid 2‐related factor (Nrf2) in vitro and vivo. Mechanistically, Sch enhances the expression of Nrf2 by regulating the degradation of Nrf2. Further, Sch suppresses phosphorylation of P65 and its nuclear translocation, as well as the degradation of IκBα. Collectively, our findings reveal that Sch protects against OVX‐induced bone loss by suppressing ROS via Nrf2.

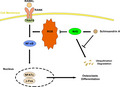

## 1 INTRODUCTION

The activity of osteoclasts (OCs) and osteoblasts (OBs) is regulated precisely in the development of skeletal system.^1^, ^2^ Some disease such as osteoporosis, rheumatoid arthritis and Paget's disease are correlated with over‐activation of OCs.^3^ The differentiation and formation of mature osteoclasts depend on the two necessary signals: macrophage colony‐stimulating (M‐CSF) and receptor activator of NF‐kB ligand (RANKL). M‐CSF promotes the proliferation and survival of osteoclast precursors by activating the AKT and ERK1/2 pathways,^4^ whereas RANKL activates NF‐kB and MAPKs (Erk, JNK and p38) by inducing the association of RANK and TRAF6. These kinases, in turn, promote the expression of nuclear factor of activated T cells 1 (NFATc1) and c‐Fos, the main transcription factors in osteoclastogenesis, hence activating the process of osteoclast differentiation.^1^

Reactive oxygen species (ROS), as a second messenger in the receptor‐mediated signalling cascades, has been proven to be a pivotal factor in the development of osteoclast differentiation.^5^ During osteoclastogenesis, ROS level increases because of the stimulations from RANKL and M‐CSF. These increases, which in turn, promote the differentiation and formation of osteoclasts.^6^, ^7^ Several oxidant scavengers have been proved to be an effective alternative to inhibit RANKL‐induced osteoclastogenesis by suppressing ROS.^5^ Under oxidative stress condition, Nuclear factor erythroid 2‐related factor 2 (Nrf2) regulates the transcription activity of several anti‐oxidant enzymes such as haem oxygenase‐1 (HO‐1), catalase, glutathione‐disulphide reductase (GSR), NAD(P)H quinone dehydrogenase 1 (NQO1) and γ‐glutamylcysteine synthetase (GCS).^8^, ^9^, ^10^

Nuclear factor erythroid 2‐related factor 2 (Nrf2) is a member of cap‘n'collar basic leucine zipper family, which functions as a transcriptional factor regulates the expression of several anti‐oxidant and phase II detoxifying enzymes, reducing the production of ROS.^9^ Overexpression of Nrf2 could inhibit osteoclastogenesis in *vitro* and *vivo*, whereas Nrf2 deficiency promotes this process.^9^, ^11^, ^12^

Schisandrin A, a dibenzocyclooctadiene lignan extracted from Schisandra chinensis (Turcz.) Baill, has attracted attention for its anti‐oxidant and anti‐inflammatory effect recently.^13^, ^14^ However, little is known about the effects of schisandrin A on the differentiation of osteoclasts.

In the present study, we investigated the effects of schisandrin A on osteoclastogenesis using a RANKL‐induced osteoclast differentiation model in vitro, and an ovariectomy‐induced osteoporosis C57BL/6 mice bone loss model in vivo. Sch decreases intracellular ROS production by increasing the expression of Nrf2, which in turn inhibited the RANKL‐induced NFATc1 and NF‐kB signalling pathway, thus suppressed the differentiation of osteoclasts.

## 2 MATERIALS AND METHODS

### 2.1 Ethics statement

The animal experiments in this study were approved by the Animal Ethics Committee of Shanghai Pudong Hospital, in accordance with the National Institutes of Health (NIH) Guide for the animal treatments of Laboratory Animals.

### 2.2 Reagents

The αMEM ( alpha modification of Eagle's medium), foetal bovine serum (FBS) and DMEM were purchased from Thermo Fisher Scientific. Penicillin/streptomycin was obtained from Beyotime Institute of Biotechnology (Beyotime, Shanghai China). Schisandrin A was obtained from MCE (MedChemExpress) and was dissolved in DMSO (0.5%w/v) at the concentration of 1 mmol/L stock solution and stored at refrigerator in −20°C. The DMSO concentration was below 0.4% to the culture medium. For the animal part, Sch was diluted in corn oil before injection into C57BL/6 mice. CCK‐8 reagent was purchased from Dojindo Molecular Technologies. Primary antibodies of Cathepsin K, NFATc1, CTR, MMP‐9, TRAcP catalase and β‐actin were purchased from Proteintech Group (Rosemont, Inc). Primary antibodies of Nrf2, HO‐1, IκBα, p‐IκBα, p65, p‐p65 and c‐Fos were obtained from CST. Primary antibody of Nrf2 in animal part was obtained from Abcam. Primary antibody of CTSK and MMP9 were purchased from Proteintech Group (Rosemont, Inc). V‐ATPase‐D2 was purchased from Santa Cruz Biotechnology. Primary antibodies of RANKL and OPG were purchased from Abclonal. The dilution of antibody used in this study was 1:1000 unless noted. M‐CSF and RANKL were purchased from R&D Systems. pNFκB‐luc was obtained from Beyotime. siRNA was purchased from GenePharma. All the chemicals and reagents we used in this study were of analytical grade.

### 2.3 BMMs isolation and differentiation

Primary mouse bone marrow‐derived macrophage cells were isolated from 6‐week‐old C57BL/6 mice as described previously.^15^ Briefly, first, according to the procedures approved by the Animal Ethics Committee of Shanghai Pudong Hospital, mice were euthanized. After that, mice were executed and the femoral was harvested. Cells were harvested in a sterile circumstance. Then, the cells were cultured in complete culture medium with 20 ng/mL M‐CSF for about 3 days. For the osteoclast (OC) differentiation, (bone marrow macrophages) BMMs were cultured at the density of 8 × 10^3^/well in 96‐well plates with induction medium containing 20 ng/mL M‐CSF and 50 ng/mL RANKL. Induction culture medium was changed every 2‐3 days until OCs formed. Then, OCs were stained by TRAcP staining (Sigma‐Aldrich) according to a previous study.^16^

### 2.4 BMSC isolation and differentiation

Primary mouse bone marrow stem cells were harvested as described previously.^17^ Briefly, mice were euthanized, executed as the same way as mentioned above. BMSCs were cultured in the DMEM containing 1% penicillin/streptomycin, 10%FBS. The cells were passaged until confluence. For the osteoblast differentiation, BMSCs were cultured and induced in the osteogenic medium (Beyotime, Shanghai China). ALP staining (Beyotime Biotechnology) was performed at day 7. Alizarin red staining (Sigma‐Aldrich) was carried out at day 14.

### 2.5 Cell viability assay

CCK‐8 assay was used to detect the cell viability according to the manufacturer's protocol. In brief, BMMs cells were seeded into 96‐well plates at different concentrations of schisandrin A (0, 50, 100, 150, 200 and 400 μmol/L) containing 20 ng/mL M‐CSF. After 24, 48 and 72 hours of incubation, the cells were tested. Cell viability was then measured by a microplate reader at the absorbance of each well at 450 nm. The same method was used to detect the cell viability of BMSCs cells as well.

### 2.6 In vitro BMM apoptosis assay

Bone marrow macrophages were cultured with 20 ng/mL M‐CSF and 50 ng/mL RANKL for 2 days; all the cells were examined by Annexin V assay kit (Phoenix Flow Systems). Cells were analysed by using the flow cytometer (BD Biosciences) as previously described.^16^

### 2.7 Rhodamine‐phalloidin staining

Bone marrow macrophages were seeded into 24‐well plates until mature OCs were formed in the stimulation of 20 ng/mL M‐CSF and 50 ng/mL of RANKL. Different concentrations of Sch were added into culture medium during the development of OCs. After 7 days of culture, Rhodamine‐phalloidin staining was performed according to the manufacturer's protocol (UE, Suzhou, China). OC nuclei were stained by DAPI. Multi‐nuclei cells (nuclei > 3) were identified as osteoclasts, and the number of nuclei was calculated.

### 2.8 Hydroxyapatite‐coated plate resorption assay

Osteo Assay surface plates (Corning) were used for the detection of resorptive test as study reported.^16^, ^17^ In brief, when mature OC formed, the cells were removed and then the disc was washed with distilled water. The area of resorption pits was measured by ImageJ software (National Institutes of Health).

### 2.9 Quantitative real‐time polymerase chain reaction (qRT‐PCR)

Quantitative real‐time polymerase chain reaction (qRT‐PCR) was used to quantify the mRNA expression of NFATc1, c‐Fos, MMP9 and TRAcP. Briefly, RNA from different groups was harvested according to the TRIzol reagent (Invitrogen) protocol. Then, they were reverse transcribed to cDNA. The mRNA expression levels were assessed by real‐time PCR system (Applied Biosystems). The specific primers we used are according to a previously report unless otherwise noted.^11^, ^12^, ^18^, ^19^ The primers are listed in Table S1.

### 2.10 ROS production assay

ROS assay kit (Beyotime) was used to detect the intracellular ROS levels. Briefly, cells were pre‐treated in different conditions of Sch for 24 hours with 50 ng/mL RANKL. Then, they were tested by a fluorescent probe (1:1000) and incubated at incubator for 30 mins after washed and centrifugation carefully. The fluorescence of DCF was measured using a laser scanning microscopy (Nikon). And the mean fluorescence intensity was quantitative analysed by ImageJ software (National Institutes of Health).

### 2.11 Immunofluorescence

All cells were pre‐treated with Sch for 24 hours and then stimulated by RANKL 50 ng/mL for 30 minutes, followed by a fixation with paraformaldehyde for 10 minutes and blocked with 5% goat serum for 1 hour. Then, cells were incubated with anti‐p65 antibody (CST, 1:200) overnight at 4°C and with a Cy3‐conjugated secondary antibody (Beyotime, 1:500) for 1 hour the next day. Images were acquired with a Zeiss LSM T‐PMT confocal microscope (Zeiss).

### 2.12 Western blotting

Cells were treated under different conditions. Then, the proteins from each group were harvested using RIPA lysis buffer (Sigma‐Aldrich) according to the manufacturer's protocol. Briefly, cells with different culturing conditions were washed with cold clean PBS three times for 1 minute each. RIPA lysis buffer containing protease and phosphatase inhibitors were used to extract cellular proteins. Proteins were separated by SDS‐PAGE and were then transferred to PVDF membranes and probed with specific primary antibodies and secondary antibodies. Electrochemical luminescence reagent (Millipore) was used to visualize the bands. The grey level of bands was quantified using ImageJ software (National Institutes of Health).

### 2.13 Cell transfection

Bone marrow macrophages were transfected with siRNA using Lipofectamine 3000 (Invitrogen) according to the instructions. Briefly, cells were seeded in 6‐well plates at the density of 2 × 10^4^ cells/well the day before transfection. The next day, cells were transfected with 20 nmol/L siRNA. Six hours later, the medium was replaced by culture medium. After 48 hours, the transfection efficiency was tested by fluorescence microscope. Cells were then harvested 72 hours later for Western blotting and qPCR to assess the expression of Nrf2. Transfected BMMs were cultured in the OC induction medium in the presence of Sch.

There are three siRNA sequences be designed. We selected the most effective one according to the results of qPCR and Western blot. The most effective sequence of Nrf2 siRNA is in Table S2.

### 2.14 NF‐κb luciferase reporter assays

NF‐κb dependent luciferase reporter assays were used as previous study to detect the effect of Sch on RANKL‐induced BMMs OC differentiation.^12^ Briefly, BMMs were cultured in 24‐well plates, and then, they were transiently co‐transfected with NF‐κB‐Luc and pRL‐TK plasmid (Promega, WI, USA) as previously reported.^12^ Lipofectamine 3000 (Invitrogen) was used for the control group.

### 2.15 Ovariectomy C57BL/6 model

Six‐week‐old female C57BL/6 mice were purchased from the Slack (Shanghai, China) and randomly divided into three groups (SHAM (PBS) group, OVX group and OVX + Sch group, n = 5). The surgical procedure was according to our previous study.^19^ Briefly, animals were anaesthetized by 5% chloral hydrate. Small incisions were made to expose the ovaries. Ovaries and oviducts of the mice in OVX group and OVX + Sch group were removed. All of the mice were allowed to recover for another 48 hours after surgery. Then, the SHAM and OVX group was injected by PBS, and OVX + Sch group was injected by Sch with 100 mg/kg body weight every other day. The solvent we used of schisandrin A in our study was according to the protocol provided by MCE. Briefly, we add each solvent one by one, 10% DMSO followed by 90% corn oil, and then, it was diluted by normal saline. All of the animals were sacrificed after 6 weeks of interventions. Inflammatory factors from blood samples were tested by ELISA. Femur bones were collected for micro‐CT and further histological analysis.

### 2.16 Measurement of mice body and uterus weight

Body weight of C57BL/6 mice from each group was measured every week since OVX mice model established. The week of OVX model being established was marked as week 0. All of the animals were sacrificed after 6 weeks of interventions. Uterus weight from each group was measured at the end of study. All of the data we collected during this process were recorded and calculated by GraphPad.

### 2.17 Histological analysis

The femurs of mice in each group were fixed in 4% formaldehyde at room temperature and decalcified in 10% tetrasodium‐EDTA aqueous solution. After carefully embedded with paraffin, samples were sectioned by microtome (Leica, Germany) in a thickness of 4 μmol/L. Next, haematoxylin and eosin (H&E) staining and TRAcP staining were carried out to evaluate the histological changes of each group. TRAcP staining was applied to calculate OC numbers. H&E staining was scanned by the Aperio Scanscope. Bone histological parameters were calculated by ImageJ software. For Nrf2 immunofluorescence staining, the samples were pre‐treated with 10% goat serum. Then, the cells were incubated with the primary antibody (1:200) overnight at 4°C. Secondary antibody conjugated with Alexa Fluor 488 (1:500) was used the next day. DHE staining was performed according with the protocol previously reported.^20^ Briefly, fresh bone femurs were harvested and then fixed in 4% paraformaldehyde solution at 4°C for 4 hours immediately. Next, decalcification using EDTA was carried out overnight. Then EDTA was replaced by cryoprotective solution the next morning. All of the tissues were in cryoprotective solution for another 24 hours. Finally, the bone sections were embedded and frozen. A thickness of 5 μm sections was made. Nuclei were stained with DAPI. Five random areas from each group were quantified and analysed by ImageJ software.

### 2.18 Liver histological evaluation

Liver tissue was fixed by paraformaldehyde for 24 hours and then be embedded in paraffin for H&E staining. Liver tissue damage was evaluated by a histological score from Histological Activity Index (HAI).

### 2.19 Micro‐CT scanning

The fixed femurs were analysed by high‐resolution μCT (SkyScan, Antwerp, Belgium). Protocol of scanning was according to the previous study.^16^ In brief, left femurs of mice were collected and scanned with the following parameters: source voltage, 50 kV; source current, 450 μA; AI 0.5 mm filter; pixel size 9 μm; and rotation step, 0.4 degree. NRecon software (Bruker microCT, Kontich, Belgium) was used to reconstruct the images followed by micro‐CT scanning. Parameters were set as follows: ring artefact correction, 8; smoothing, 2; and beam hardening correction, 30%. The constant threshold was set as 80‐255. Bone volume fraction (BV/TV), trabecular thickness (Tb. Th) and trabecular number (Tb. No), trabecular spacing (Tb. Sp) were measured by the program CTAn (Bruker micoCT).

### 2.20 Enzyme‐linked immunosorbent assay (ELISA)

Bone marrow macrophages were pre‐treated in different concentrations of Sch for 24 hours with 50 ng/mL RANKL. The levels of nitric oxide synthase (NOS2), thioredoxin (TRX1) and glutathione reductase (GSR) in the supernatant of BMMs and the serum levels of TNF‐a, IL‐1β, IL‐6, C‐telopeptide 1 (CTx‐1), ALT, AST, BALP and OCN of mouse were measured by ELISA kit (FineTest, China), according to the manufacturer's instructions. The absorbance (450 nm) of each sample was detected on a standard automatic microplate reader.

### 2.21 Osteoblast staining

Primary BMSCs were cultured in DMEM culture medium; then, they were seeded and cultured in 96‐well plates until 90% confluence. Osteogenic medium (Beyotime, China) was used to induce osteoblasts. After 14 days, alkaline phosphatase activity and mineralization activity were detected by alkaline phosphatase staining (ALP) and Alizarin red staining according to the manufacturer's instructions (Beyotime).

### 2.22 Statistical analysis

All experimental data were presented as the mean ±SD (n ≥ 3) from three or more independent experiments. GraphPad Prism (version 7, GraphPad Software) was used to assess the data. Student's t test or Mann‐Whitney test was used in statistical analysis. One‐way ANOVA with Turkey's post hoc test was used for analysing the differences between groups. LSD t test was applied when data needed to be compared with control in multiple groups. Bonferroni's correction was performed for multiple comparisons in data. *P < .05 and **P < .01 were considered to be significant.

## 3 RESULTS

### 3.1 Schisandrin A decreases OVX‐induced bone loss in vivo

**FIGURE 1:**
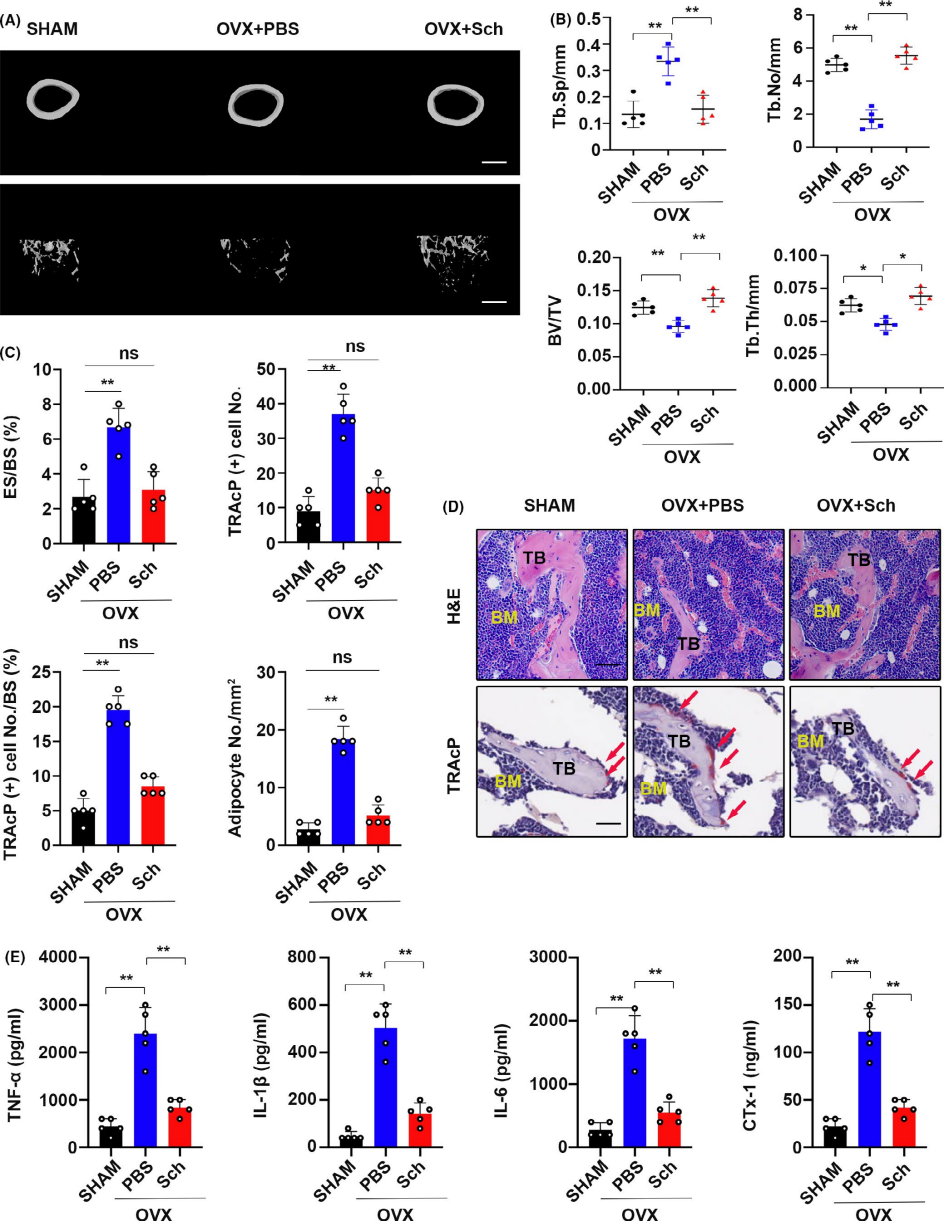
Sch reduced OVX‐induced bone loss in vivo. A, Representative micro‐CT of trabecular (Tb) and cortical (Ct) bone from each group (n = 5 per group). Scale bar = 500 μm. B, Quantification of trabecular separation (Tb.Sp), trabecular number (Tb.N), bone volume/tissue volume (BV/TV) and trabecular thickness (Tb.Th). C, Quantitative analysis of the eroded surface/bone surface (ES/BS), TRAP‐positive cell numbers from five different versions, TRAP‐positive cell number/bone surface and the adipocyte number per field. D, H&E staining was performed to test the bone loss; TRAcP staining was used to detect OCs. Scale bar = 20 μm. TB, trabecular bone; BM, bone marrow; osteoclasts are indicated by red arrows. E, Serum levels of TNF‐a, IL‐1β, IL‐6 and C‐telopeptide 1 (CTx‐1) in mice from each group were determined by ELISA. The data are shown as means ± SD (n = 5). ns, no significance. *P < .05, **P < .01

Ovariectomized‐induced osteoporosis mice model was established to explore the effect of Sch in vivo, the femur was isolated to undergo a micro‐CT. Extensive bone loss was observed in the OVX group; however, Sch‐treated group showed no significant bone loss when compared with SHAM group (Figure 1A). Parameters of trabecular separation (Tb.Sp), trabecular number (Tb. No.), trabecular thickness (Tb.Th,) and bone volume/ tissue volume (BV/TV) were measured and compared with each other in this section. Increases in Tb.N, BV/TV and Tb.Th were observed in Sch group, whereas Tb.Sp decreases compared to that of OVX + PBS group (Figure 1B). Histological analysis using H&E staining, Sch could significantly decrease OVX‐induced bone loss in vivo. Further, TRAcP staining of bone slide was carried out to evaluate the effect of Sch in the OC differentiation. Five representative paraffin bone slides were selected to undergo a TRAcP staining; then, the TRAcP‐positive cells were measured randomly from five different versions. Large number of osteoclasts in OVX + PBS group was observed; however, little was shown in Sch group. These osteoclasts, in turn, resulted in a decrease of eroded surface/ bone surface and TRAcP‐positive cells/ bone surface in the Sch group (Figure 1C,D). ELISA assay was carried out to detect the inflammatory and osteoporosis‐related factors in vivo, all of which showed a lower level in Sch group compared with PBS + OVX group (Figure 1E). Effects of Sch on mice in vivo such as body weight, uterus weight and liver function also had been investigated (Figure S3). These results indicated that schisandrin A may have an effect on the mitigation of OVX‐induced bone loss.

### 3.2 Sch suppresses ROS production and enhances the expression of Nrf2 in vivo

**FIGURE 2:**
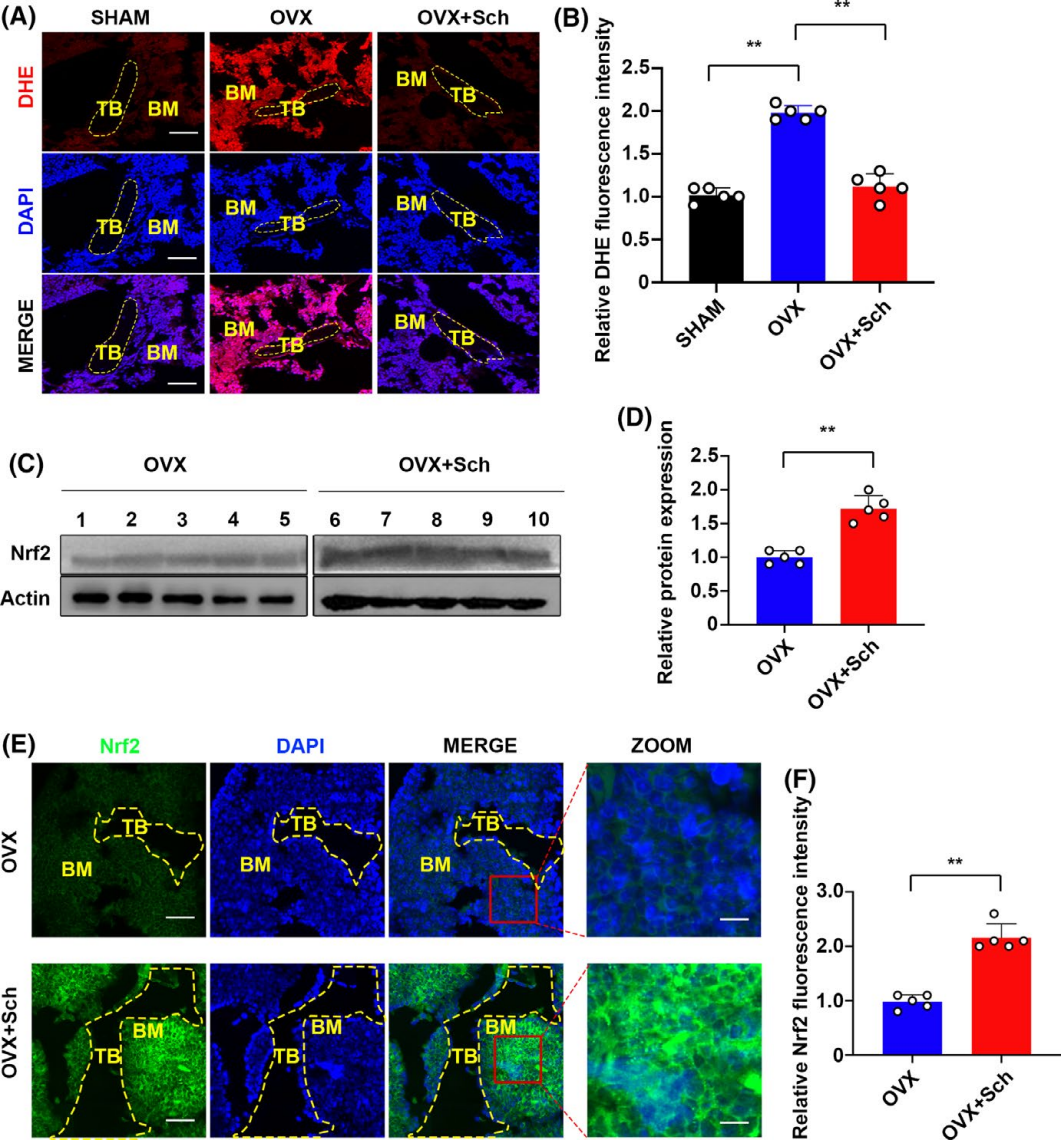
Sch reduced ROS production and enhanced the expression of Nrf2 in vivo. A, Representative images of bone cryosections showing DHE fluorescence in different groups. Scale bar = 200 μm. DHE, dihydroethidium, B, Quantification of DHE fluorescence intensity of each group (n = 5 per group). C, Proteins were isolated from bones from each group, and Nrf2 expression was investigated using Western blotting. OVX + PBS group (1,2,3,4,5) and the OVX + Sch group (6,7,8,9,10). Nrf2: Nuclear factor erythroid 2‐related factor 2. D, Quantification of Western blotting bands. The data are shown as means ± SD (n = 5). E, Immunofluorescence staining was performed to detect the expression of Nrf2. Scale bar = 200 μm. Nuclear translocations of Nrf2 were observed in the zoom pictures. Scale bar = 100 μm. F, Quantitative analysis of Nrf2 immunofluorescence intensity. TB, trabecular bone; BM, bone marrow; DAPI, 4,6‐diamidino‐2‐phenylindole; DHE, dihydroethidium; ROS, reactive oxygen species; Nrf2: nuclear factor erythroid 2‐related factor 2. The data are shown as means ± SD (n = 5). ns, no significance. *P < .05, **P < .01

Next, we investigated the underlying mechanisms that possibly affect the OC differentiation in OVX‐induced model. Emerging studies suggest that ROS is essential in the differentiation of OC.^5^, ^6^, ^16^, ^21^ Hence, ROS production in vivo was detected by DHE fluorescence staining using cryosections of femur bone slides as previously described.^17^ Interestingly, Sch group showed a significant decrease in the matter of fluorescence intensity, whereas OVX group showed an increase of that due to ovariectomy (Figure 2A,B). As a key transcriptional factor regulates the expression of several anti‐oxidant enzymes, Nrf2 is a critical linchpin in this process.^9^ Therefore, we explore whether Nrf2 expression was enhanced in Sch group compared to OVX group in vivo. As shown in Figure 2C, total protein of proximal tibias was extracted from each group and Nrf2 expression was investigated using Western blotting. Enhancement of Nrf2 expression was observed in Sch‐treated group. Quantitative analysis by ImageJ showed a higher protein expression compared to OVX group (Figure 2D). Further, to confirm our results from Western blot, immunofluorescence staining of Nrf2 was carried out using paraffin bone slides. Nrf2 immunofluorescence staining in Sch group showed stronger intensity than that of OVX one. Quantitative analysis of Nrf2 immunofluorescence signal intensity also confirmed our results (Figure 2E,F). Moreover, translocation of Nrf2 to nuclei was observed in the ZOOM pictures in zoom of Figure 2E.

### 3.3 Sch suppresses RANKL‐induced osteoclastogenesis in vitro

**FIGURE 3:**
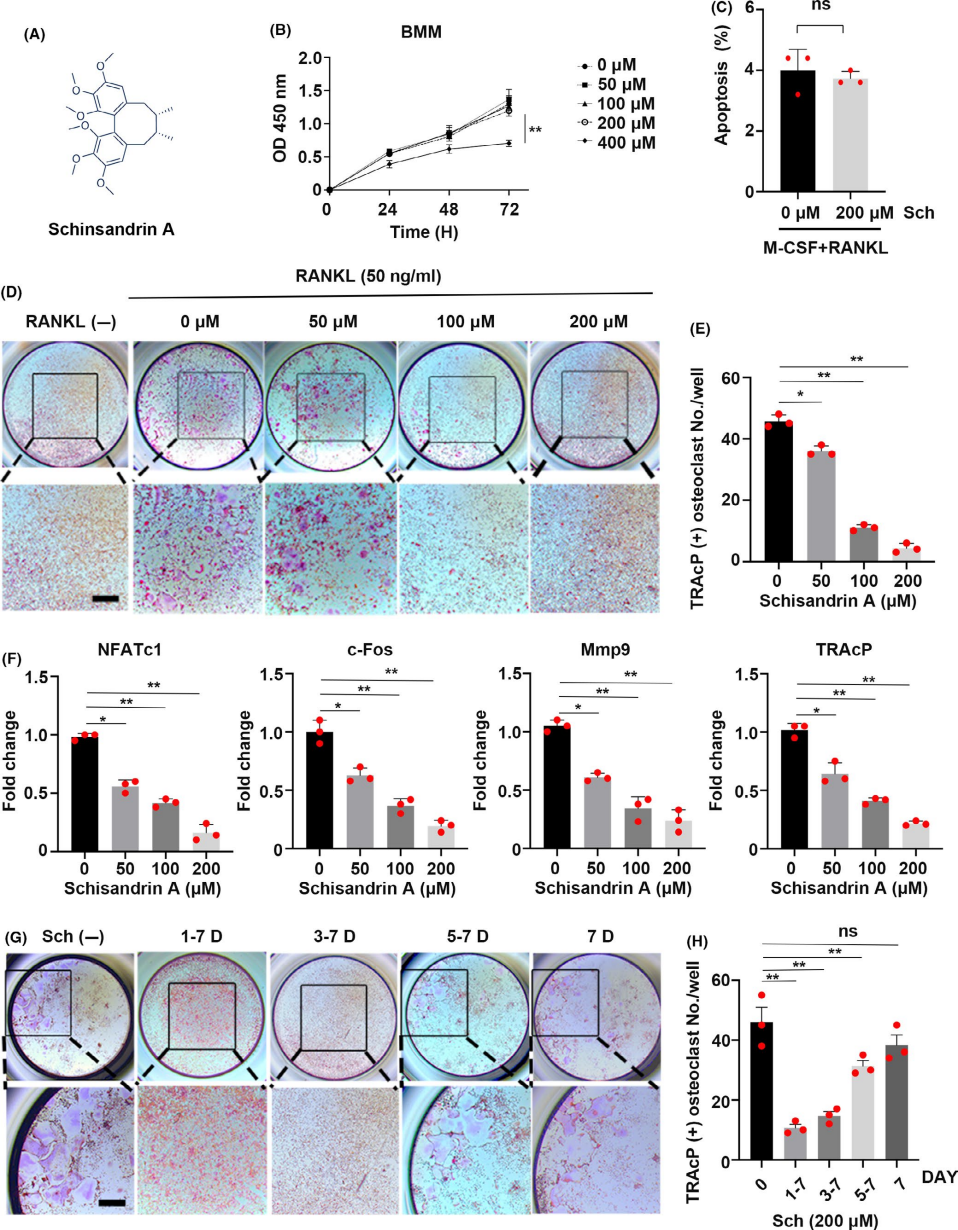
Sch suppresses RANKL‐induced osteoclastogenesis in vitro. A, The chemical structure of schisandrin A. B, Effects of Sch on BMM viability measured by CCK‐8 assay (n = 3 per group). C, Frequency of apoptotic cells of BMMs co‐cultured with 20 ng/mL M‐CSF and 50 ng/mL RANKL at the concentration of 200 μmol/L Sch for 2 d (n = 3 per group). D, Representative images of TRAcP staining showing that Sch inhibited osteoclastogenesis dose‐dependently. BMMs were stimulated with M‐CSF and RANKL in the absence or presence of different concentrations of Sch. E, Quantification of TRAcP‐positive cells per well. The data are shown as means ± SD (n = 3). BMMs, bone marrow‐derived macrophage cells. F, Quantitative qRT‐PCR of osteoclast‐specific genes including NFATc1, c‐Fos, MMP9 and TRAcP were examined in the absence or presence of different concentrations of Sch. G, Representative images of TRAcP staining showed BMMs treated with Sch 200 μmol/L for the indicated days during osteoclastogenesis. H, Quantification of TRAcP‐positive cells per well when treated with Sch in different periods (n = 3 per group). The data are shown as means ± SD. ns, no significance. *P < .05, **P < .01. Scale bar = 200 μm

The chemical structure of Sch is shown in Figure 3A. Fresh bone marrow macrophages (BMMs) from C57BL/6 mice were isolated using methods as described in Methods. We found that Sch had no effect on the viability of BMMs below the concentration of 200 μmol/L in the presence of 20 ng/mL M‐CSF (Figure 3B). Also, we found that Sch did not affect the viability of bone marrow stem cells (BMSCs) below 200 μmol/L (Figure S1A). Notably, the concentration of Sch at 200 μmol/L, which we would use later in our experiment, did not induce apoptosis of BMMs when co‐culture with M‐CSF and RANKL for 2 days (Figure 3C). Moreover, Sch below 200 μmol/L co‐cultured with mature OC did not induce apoptosis as well (Figure S2A). M‐CSF related receptors Mitf and TFE3 are reported to be critical in the development of osteoclasts.^22^ Sch had no effect on both receptors following a 24‐hour treatment of M‐CSF with/ without RANKL or Sch (Figure S2B). Then, BMMs were seeded in 96‐well plates and treated with both M‐CSF and RANKL in the presence of different concentrations of Sch as showed in Figure 3D. Corresponding quantitative analysis of TRAcP‐positive cells was confirmed in Figure 3E. These results demonstrated that the increasing concentration of Sch attributed to a dose‐dependant manner inhibition on osteoclastogenesis. Next, quantitative PCR of osteoclast‐specific genes including NFATc1, c‐Fos, MMP9 and TRAcP were examined to observe the underlying changes at mRNA level in different concentrations of Sch (Figure 3F). Altogether, these results showed that Sch could effectively inhibit RANKL‐induced osteoclastogenesis in a dose‐dependent manner. To explore at which period Sch functioned in the development of OC, BMMs were treated with 200 μmol/L Sch at indicated time phase (Figure 3G). Sch showed its suppressive effect at days 3‐7, rather than days 5‐7 (Figure 3H). Collectively, these results indicated that Sch inhibits RANKL‐induced osteoclastogenesis in a dose‐dependent and time‐dependent manner.

### 3.4 Sch affects podosome belt formation and inhibits osteoclast resorptive function

**FIGURE 4:**
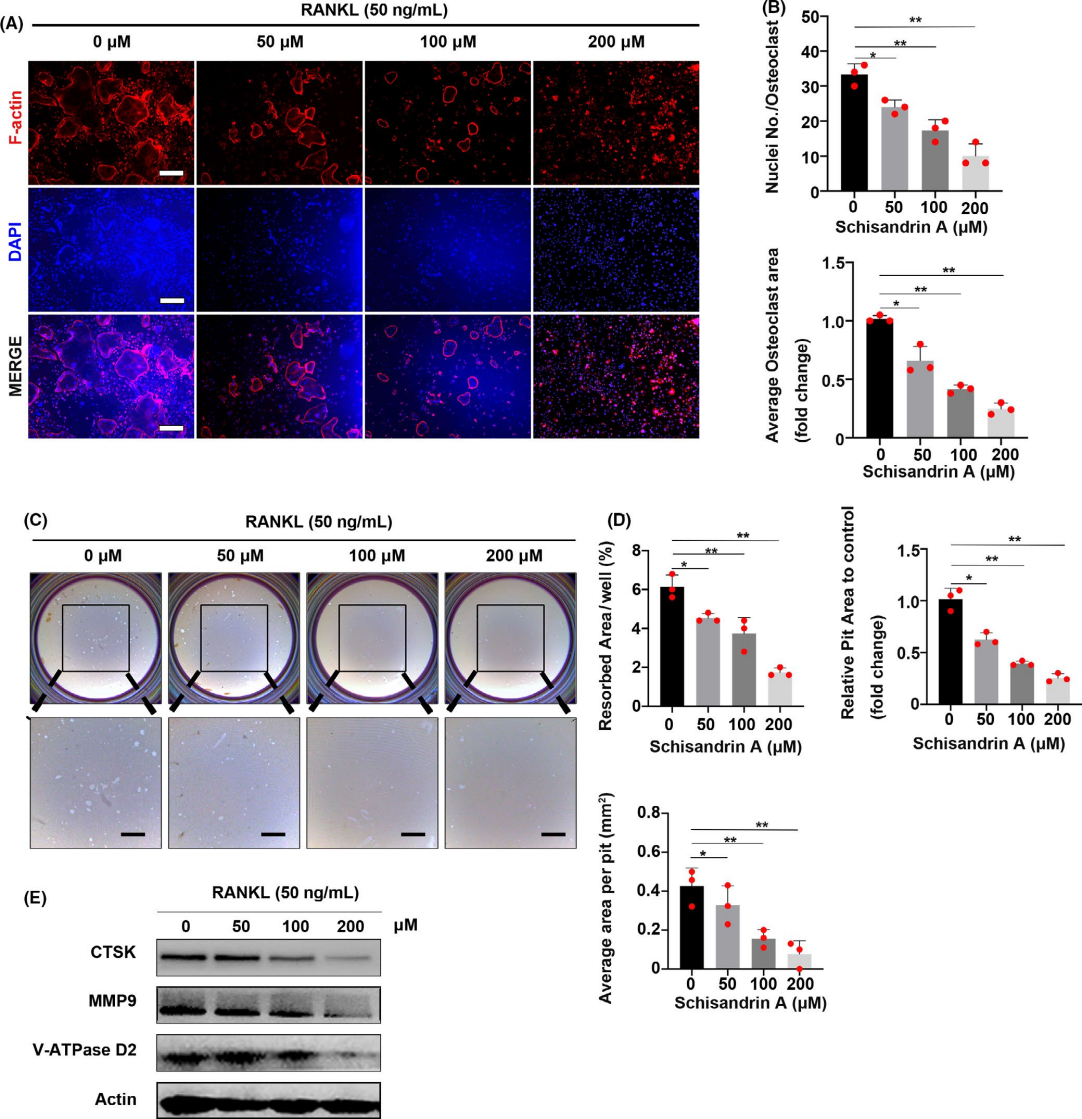
Sch affects podosome belt formation and inhibits osteoclast resorptive function. A, Representative images of podosome belt formation in OCs treated with different concentrations of Sch. Images were observed by confocal microscopy. Scale bar = 200 μm. OC, osteoclast. B, Number of nuclei per osteoclast and average OC area was quantified. C, Hydroxyapatite‐coated plates were used to detect osteoclast resorptive activity at different concentrations of Sch. Scale bar = 500 μm. D, Quantification of pit area and resorbed area. (n = 3 per group). E, Bone resorption‐related proteins CTSK, MMP9 and V‐ATPase D2 are detected by WB. An inhibition of bone resorptive effects was found in different concentrations of Sch. The data are shown as means ± SD. ns, no significance. *P < .05, **P < .01

Bone marrow macrophages were co‐cultured in different concentrations of Sch both with M‐CSF and RANKL for 7 days; then, the formed osteoclasts were stained with rhodamine‐phalloidin to investigate the morphological changes under different conditions. F‐actin ring formation was significantly inhibited at the concentration of 200 μmol/L compared to that of other groups (Figure 4A). The number of nuclei per osteoclast decreased from 36/osteoclast (Sch 0 μmol/L) to 9/osteoclast (Sch 200 μmol/L) (Figure 4B). Next, hydroxyapatite‐coated plates were used to determine whether Sch had an effect on osteoclast resorptive activity. As shown in Figure 4C, an inhibitory effect of Sch on osteoclast resorptive function was presented in a dose‐dependent manner. The concentration in 200 μmol/L group had a significant inhibitory effect compared with other groups, and quantification of average area per pit was also evaluated according to osteo assay results (Figure 4D). In addition, we investigated the bone resorption‐related proteins CTSK, MMP9 and V‐ATPase D2 by WB. An inhibition of bone resorptive effect was found in different concentrations of Sch (Figure 4E).

### 3.5 Sch reduces RANKL‐induced ROS production on osteoclastogenesis by down‐regulating TRAF6/Nox1 signalling pathway and enhances the expression of Nrf2

**FIGURE 5:**
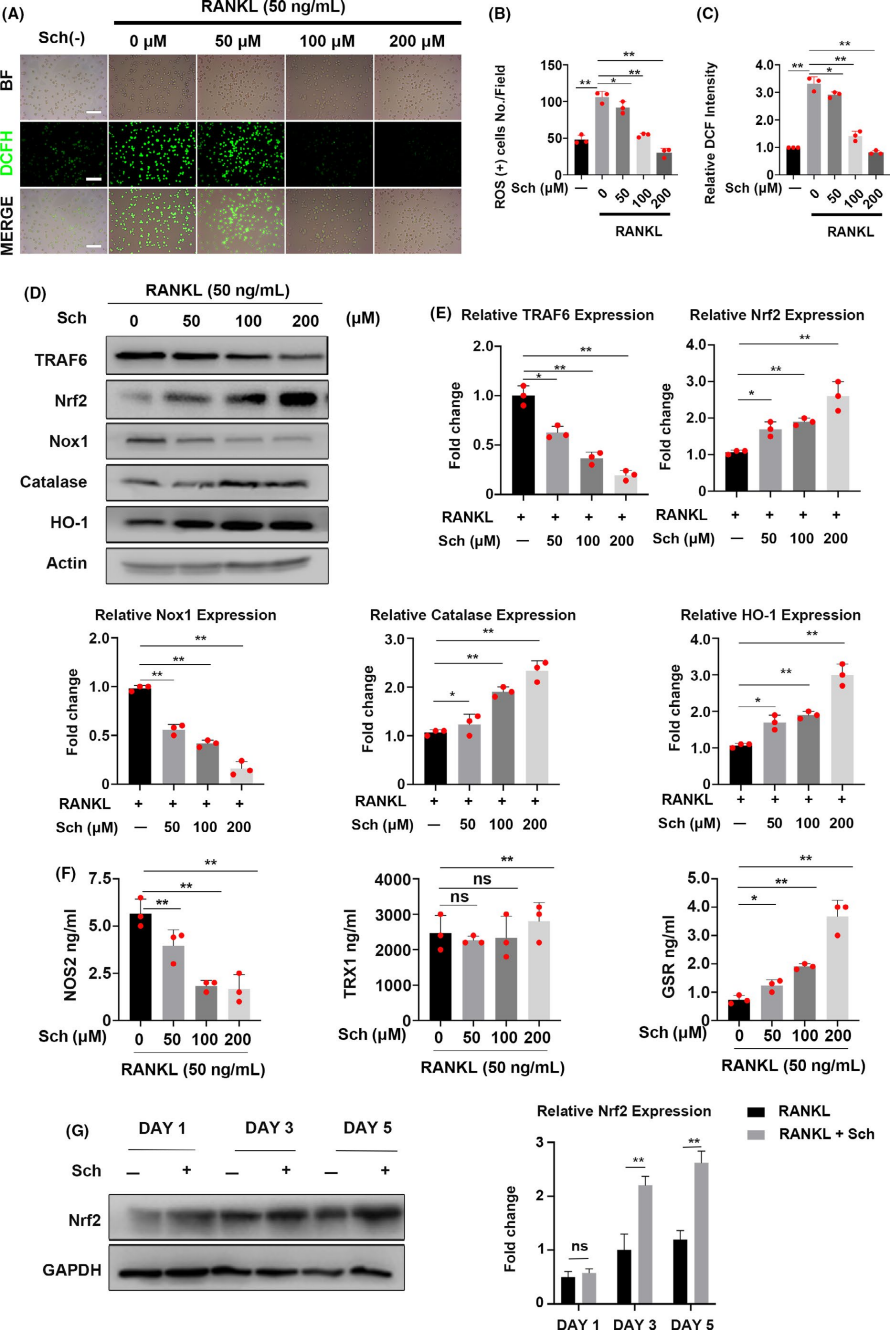
Sch reduces RANKL‐induced ROS production on osteoclastogenesis by down‐regulating TRAF6/Nox1 signalling pathway and enhancing the expression of Nrf2. A, Representative images of RANKL‐induced ROS generation in BMMs with or without pre‐treatment of Sch at different concentrations. DCFH‐DA staining was used to detect the intracellular ROS level. BF, bright field; DCFH‐DA, 2’,7’‐dichlorofluorescin diacetate; Scale bar = 200 μm. B, Quantification of the number of ROS‐positive cells per field (n = 3). C, Quantification of relative DCF fluorescence intensity averaged on cells of each well (n = 3 per group). D, Western Blot images of the effects of Sch on TRAF6, Nrf2, Nox1, catalase and HO‐1 expression. BMMs were stimulated by RANKL (50 ng/mL) in the absence or presence of Sch (0, 50, 100, 200 μmol/L) for 2 d, and then, proteins were harvested for Western blot. E, Quantification of the fold change of band intensity from each band relative to actin (n = 3 per group). F, BMMs were pre‐treated in different concentrations of Sch for 24 h with 50 ng/mL RANKL. Levels of anti‐oxidant enzymes in osteoclast culture following Sch treatment were analysed by ELISA. G, BMMs were co‐cultured with Sch 200 μmol/L at indicated day. Total protein of BMMs was harvested to test the Nrf2 expression. Sch enhanced Nrf2 expression. The data are shown as means ± SD. ns, no significance. *P < .05, **P < .01

As reactive oxygen species (ROS) plays a crucial role in osteoclastogenesis,^5^ we investigated the production of ROS using DCFH‐DA staining (Figure 5A). ROS‐positive cells per field were underwent a quantitatively analysis by ImageJ software (Figure 5B). The intensity of DCF fluorescence in 200 μmol/L was significantly reduced compared to that of other groups (Figure 5C). Altogether, DCFH‐DA staining indicated that Sch could effectively reduce the RANKL‐induced ROS production during the development of osteoclastogenesis. Studies have shown that TRAF6/Nox1 signalling pathway acted as a major contributing factor in RANKL‐induced ROS production.^5^ Hence, Western blot was used to investigate the expression of TRAF6, Nox1 and ROS‐related enzymes including Nrf2, Catalase as well as HO‐1. Sch could inhibit the expression of TRAF6 and Nox1 in a dose‐dependent manner. Moreover, this compound could effectively enhance the expression of anti‐oxidant enzymes such as Nrf2, catalase and HO‐1 (Figure 5D). Quantitative analysis by ImageJ software corroborated the result of Western blot of each protein, respectively (Figure 5E). The levels of anti‐oxidant enzymes in osteoclast culture following Sch treatment including nitric oxide synthase (NOS2), thioredoxin (TRX1) and glutathione reductase (GSR) in the supernatant of BMMs were measured by ELISA kit (FineTest, China). ELISA results demonstrated that Sch had an anti‐oxidant effect when stimulated by RANKL (Figure 5F). Nrf2 functions as a transcriptional factor which regulates the expression of several anti‐oxidant and phase II detoxifying enzymes, hence decrease the production of ROS.^9^, ^11^, ^12^ We then tested whether this compound could enhance the expression of Nrf2, the lynchpin factor in anti‐oxidant system, during osteoclast differentiation. As shown in Figure 5G, BMMs were co‐cultured with Sch at indicated day, after which total protein of BMMs was harvested to test the Nrf2 expression. Sch enhanced Nrf2 expression as quantitative analysis also corroborated our results.

### 3.6 Sch suppresses the degradation of Nrf2 by enhancing its stability

**FIGURE 6:**
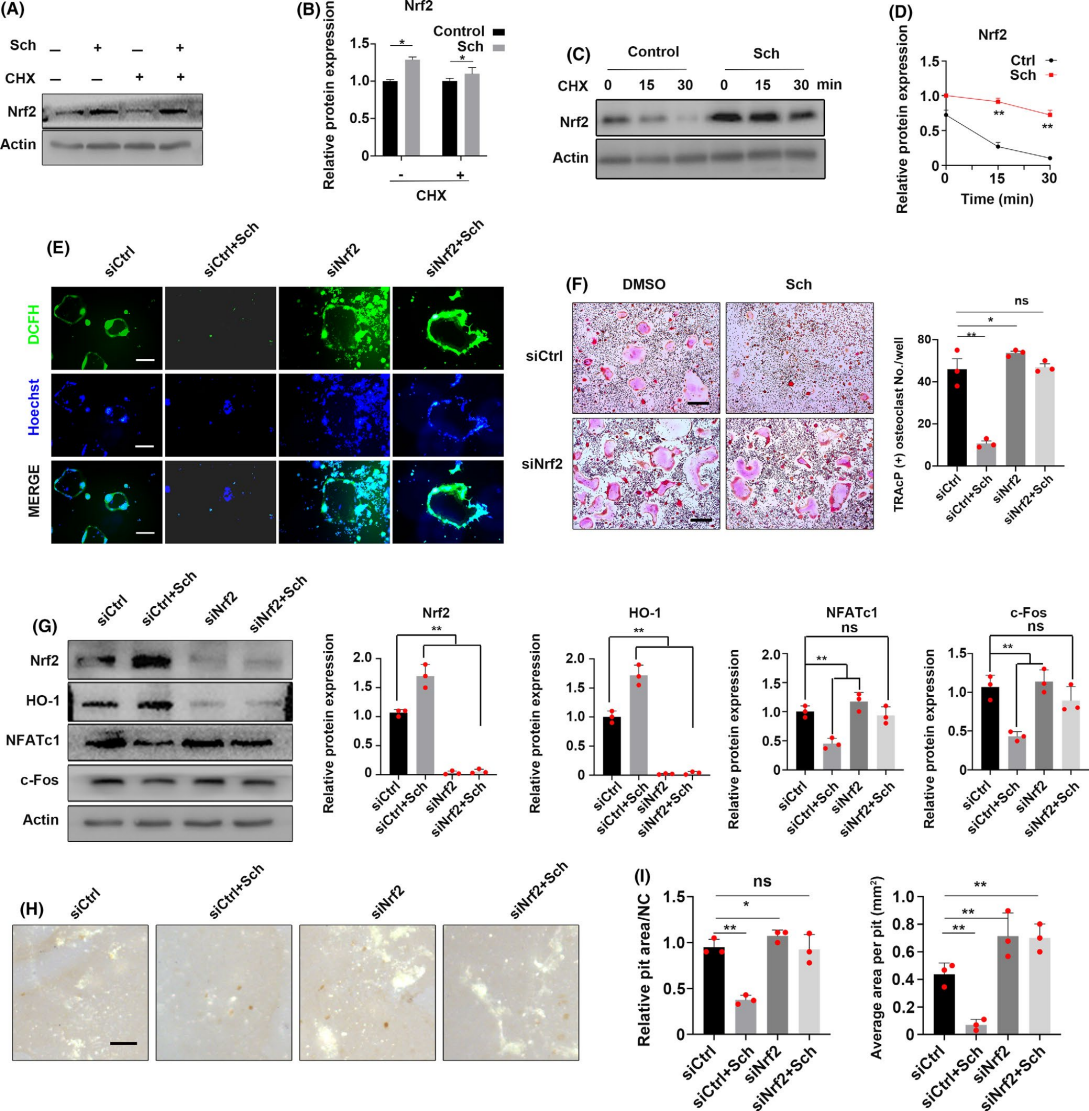
Sch suppresses the degradation of Nrf2 by enhancing its stability. Sch inhibited osteoclastogenesis by Nrf2. A, BMMs were pre‐treated with CHX for 6 h. Then, they were stimulated with Sch for 12 h. The expression of Nrf2 was analysed by Western blotting. B, Quantification of relative Nrf2 expression. C,D Degradation rate of Nrf2. BMMs were treated with CHX for the indicated time following the stimulation of Sch. The expression of Nrf2 was measured by Western blotting. E, RNAi was used to explore the role of Nrf2 and ROS in osteoclasts. Osteoclasts in siNrf2 group showed a larger area as well as higher fluorescence intensity of DCFH compared to those of other groups. Moreover, Sch could hardly rescue siNrf2‐mediated osteoclastogenesis. F, BMMs were transfected with siRNA against Nrf2 to explore its role in osteoclast differentiation. TRAcP staining was performed to detect OCs, and quantification of TRAcP‐positive cell numbers per well. Scale bar = 200 μm. G, Nrf2 knockdown could enhance the expression of NFATc1 and c‐Fos. Sch could hardly rescue the siNrf2‐mediated upregulation of NFATc1 and c‐Fos. H,I, Hydroxyapatite‐coated plates were used to detect siNrf2‐mediated OC functions. Scale bar = 200 μm. The data are shown as means ± SD. ns, no significance. *P < .05, **P < .01

Previously we have demonstrated that Sch could enhance the expression of Nrf2 in vivo and in vitro; then, we want to explore the underlying mechanism that how this compound functioned. Nrf2 is degraded rapidly by proteasome through E3 ubiquitin ligase substrate adaptor in cytoplasm. In addition, Nrf2 is a protein of low abundance with a limited half‐life of only about 15‐40 minutes. Firstly, we want to explore whether Sch could regulate the stabilization of Nrf2. At the beginning of each phase, cyclohyximide (CHX, 30 μg/mL) was added for 6 hours to block the translation before the onset of osteoclastogenesis. Then, the medium containing CHX was replaced by 200 μmol/L Sch and stimulated with another 12 hours, after that total proteins were extracted for Western blot. We found that Sch could inhibit the degradation of Nrf2 in vitro (Figure 6A), which was confirmed by quantitative analysis from Western blot (Figure 6B), implying that this compound may have an effect on the stability of Nrf2 protein. Accordingly, we wanted to investigate the degradation rate of Nrf2 under different conditions. Given the fact that CHX functioned immediately once being added into the culture medium, and Nrf2 was a protein of low abundance and degraded very fast in cytoplasm, BMMs were pre‐treated by Sch for 24 hours and then co‐cultured with CHX or not at indicated times. Interestingly, this compound could delay the degradation rate of Nrf2, for it appeared to be more stable at 15 and 30 minutes when compared with control group (Figure 6C,D).

### 3.7 Sch inhibits osteoclastogenesis by regulating ROS via Nrf2

Next, we investigated the connection between Nrf2, ROS and osteoclasts. RNAi of Nrf2 was generated by siRNA in BMMs. The inhibition of Nrf2 promoted ROS in osteoclast and thus increased osteoclastogenesis. Osteoclasts in siNrf2 group showed a larger area as well as higher fluorescence intensity of DCFH when compared to those of other groups (Figure 6E). Moreover, Sch could hardly rescue the siNrf2‐mediated osteoclastogenesis, implying that Nrf2 could be the potential target of schisandrin A. We then wanted to explore the effect of Nrf2 in RANKL‐induced in the development of OC differentiation. BMMs were transfected with siRNA against Nrf2 to explore its role in osteoclast differentiation. Surprisingly, we found that BMMs treated with siNrf2 showed more TRAcP‐positive cells compared with siControl group, indicating that Nrf2 knockdown may contribute to OC differentiation (Figure 6F). Moreover, Sch could hardly rescue the effect of siNrf2‐mediated osteoclastogenesis, suggesting Sch targeted Nrf2 to suppress osteoclastogenesis in vitro. Next, BMMs were harvested from each group to test the expression of Nrf2, HO‐1 as well as the key transcriptional factor of osteoclast‐related transcriptional factor NFATc1 and c‐Fos. We observed that siNrf2 could enhance the expression of NFATc1 and c‐Fos, whereas Sch partially rescued the siNrf2‐mediated upregulation of NFATc1 and c‐Fos (Figure 6G). In addition, siNrf2‐mediated OC function was tested by hydroxyapatite‐coated plate resorptive experiment, quantification of average area per pit was also evaluated (Figure 6H,I).

### 3.8 *Sch suppresses RANKL‐induced NF‐κB signalling* in vitro* by Nrf2*

**FIGURE 7:**
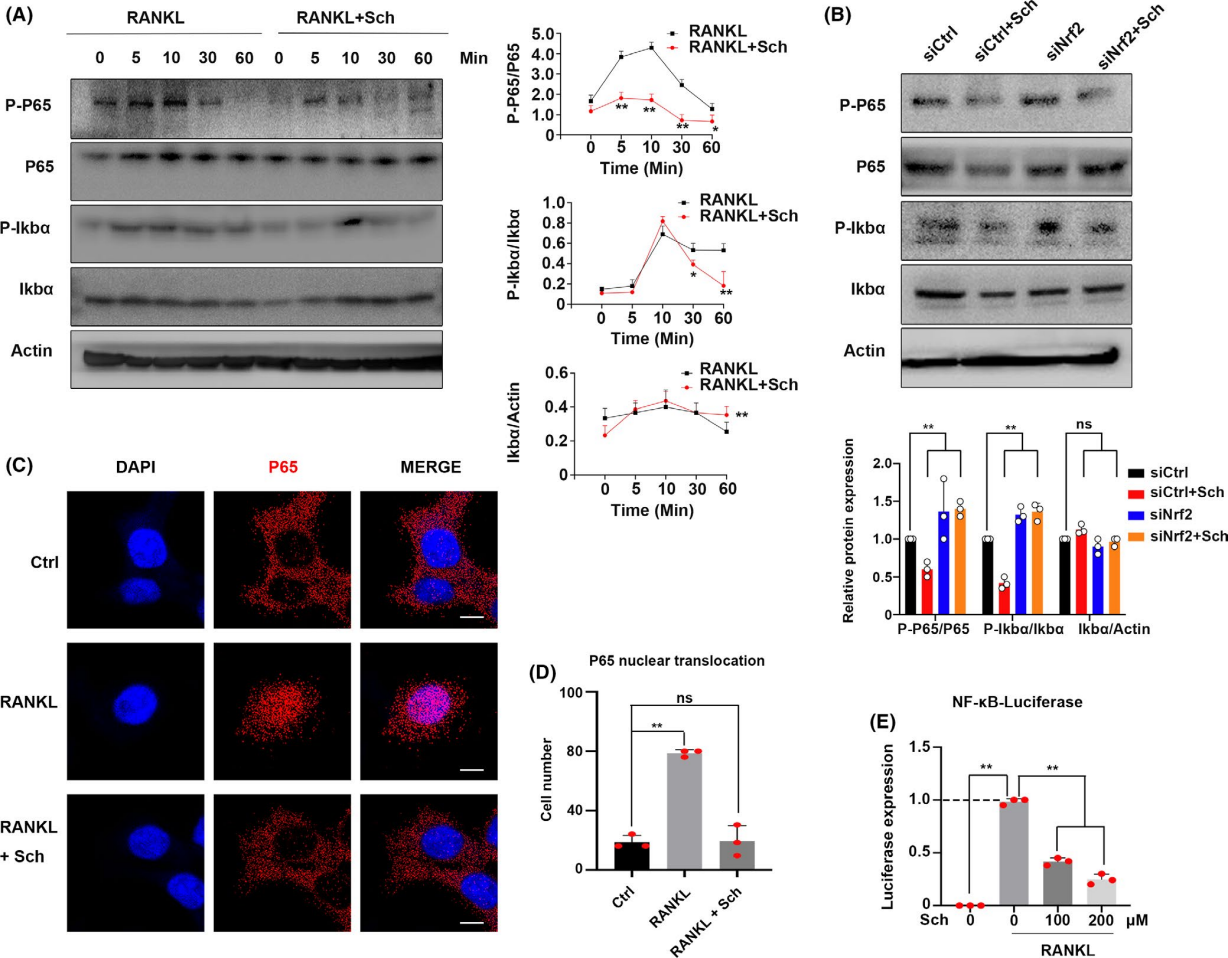
Sch suppresses the RANKL‐induced NF‐κB signalling in vitro. A, Bone marrow‐derived macrophage cells (BMMs) were pre‐treated with Sch for 24 h before stimulating by RANKL. RANKL‐induced expression and phosphorylation of IκBα as well as P65 were measured by Western blotting. B, RNAi of Nrf2 was generated by siRNA in BMMs. The phosphorylation of P65 and Iκbα at 30 mins had been detected by WB. Inhibition of Nrf2 increased the phosphorylation of P65 and Iκbα. C, D BMMs were pre‐treated with Sch for 24 h and then stimulated with RANKL for 30 mins. Immunofluorescence staining was performed to test the nuclear translocation of P65 upon RANKL stimulation in BMMs. Nuclear translocation of P65 was observed in RANKL group. Scale bar = 20 μm. E, NF‐κB luciferase assay was carried out to confirm NF‐κb transcriptional activity. BMMs were transfected with the pNFκB‐luc plasmid for 24 h and pre‐treated with Sch for 24 h before stimulating by RANKL. The data are shown as means ± SD. ns, no significance. *P < .05, **P < .01

NF‐κB signalling pathway has been proved as a lynchpin in the differentiation of OC.^19^ The phosphorylation of P65 and Iκbα had been detected. Significantly, inhibition was observed when BMMs treated with Sch at the indicated time point (Figure 7A). The degradation of Iκbα also had been investigated, and this compound showed inhibitory effect as well. Relative quantitative analysis corroborated the inhibitory effect of Sch on NF‐κB signalling pathway. Moreover, to further investigate the underlying mechanisms of Sch on NF‐κB signalling pathway, RNAi of Nrf2 was generated by siRNA in BMMs. The phosphorylation of P65 and Iκbα at 30 mins had been detected by WB. We found that inhibition of Nrf2 increased the phosphorylation of P65 and Iκbα, whereas Sch could hardly rescue the siNrf2‐mediated NF‐κB activation (Figure 7B), indicating that Sch suppresses the RANKL‐induced NF‐κB signalling by Nrf2. Immunofluorescence (IF) was performed to investigate the nuclear translocation of P65. We found this compound could effectively inhibit the translocation of P65 (Figure 7C). Cell number of P65 nuclear translocation was quantified by ImageJ software (Figure 7D). Moreover, NF‐κB luciferase reporter system was performed to detect the effect of Sch on NF‐κB transcriptional activity. We found that Sch inhibited the activity of NF‐κB transcriptional when BMMs stimulated with RANKL (Figure 7E). Additionally, osteoblast‐related histological analysis including ALP and Alizarin red S staining showed Sch had no effect on the differentiation of BMSCs (Figure S1B). Osteoblast‐related genes and proteins expression from respective mouse also had been investigated using qPCR and Western blotting, which showed no significant variance between Sch‐treated group and control group (Figure S1C,D). Moreover, Serum levels of BALP and OCN also showed no significant variance between Sch‐treated group and control group (Figure S1E). Altogether, this compound had no effect in the differentiation of osteoblast in vitro and vivo.

## 4 DISCUSSION

In this study, we proposed a novel anti‐oxidant compound schisandrin A, which had an effect on the differentiation of osteoclast by inhibiting ROS and enhancing the expression of Nrf2 in vitro and in vivo. Mechanistically, schisandrin A inhibits the degradation of Nrf2 in vitro. Nrf2 knockdown promotes the formation of OCs quantitatively and functionally. NF‐κB signalling pathway is inhibited in the schisandrin A–mediated osteoclastogenesis.

Emerging studies suggested that reactive oxygen species (ROS) is indispensable in the development of osteoporosis.^5^, ^16^, ^21^ Intracellular ROS play a second messenger role as regulator in receptor‐mediated signalling processes.^16^, ^23^ RANKL induces ROS on osteoclastogenesis, which, in turn, reinforces the activation of RANKL‐mediated signalling.^5^ Therefore, the application of anti‐oxidant compound might constitute a novel plausible strategy for the treatment of osteoclast‐related disease. Recent studies indicated that schisandrin A exhibits the anti‐oxidant activities.^13^, ^14^, ^24^, ^25^ In the present study, we found that schisandrin A had an inhibitory effect on the development of osteoporosis, as it was confirmed by Micro‐CT, H&E staining and TRAcP staining in vivo. Moreover, schisandrin A suppressed RANKL‐induced ROS production via Nrf2 in vivo. In our study, DHE staining of bone slides was used to detect ROS level, making our work completely and coherently like previously reported.^17^, ^20^ The OVX group showed an increased DHE fluorescence intensity; in contrary, schisandrin A group showed a lower intensity. Moreover, as a key anti‐oxidant transcriptional factor, Nrf2 expression in Sch‐treated group was higher than that of OVX group. In addition, we observed the nuclear translocations of Nrf2 in vivo. Altogether, our findings suggest that schisandrin A exerts its anti‐oxidant function in OVX‐induced osteoporosis model by enhancing the expression of Nrf2.

Previous studies have demonstrated that Nrf2 regulates the ROS level and determines the fate of osteoclasts.^9^, ^12^ The degradation of Nrf2 is mediated by the ubiquitin and proteasome system (UPS).^26^ Under oxidative condition, modification of cysteine residues in Kelch‐like ECH‐associated protein 1 (Keap1) leads to the release of Nrf2, resulting in the escape of Nrf2 from proteasome degradation.^27^ We first wanted to explore the mechanisms of Sch‐mediated protein stability, so we blocked the translation of Nrf2 using CHX regardless of the influence of Sch. Hence, a pre‐treatment of CHX for 6 hours was performed in BMMs, followed by another 12‐hour treatment of Sch (without CHX). Bewilderingly, WB results showed there are no differences between the CHX treatment group and control group, implying that Nrf2 degraded rapidly and completely in this process. We then optimized our plan and made some changes of stimulation conditions of CHX and Sch. Interestingly, we found that Sch could inhibit the degradation of Nrf2, which in turn demonstrated that Sch could enhance the stability of Nrf2. Nrf2 deficiency promoted the RANKL‐induced OC differentiation, whereas activating Nrf2 could inhibit this process.^9^ Our results showed that the depletion of Nrf2 using siRNA promote osteoclastogenesis like previously reported,^9^, ^11^, ^12^ indicating that Sch‐mediated Nrf2 protects osteoclast precursors (BMMs) against osteoclastogenesis in vitro. Furthermore, according to our TRAcP staining and hydroxyapatite‐coated plate resorptive assay, depletion of Nrf2 could partially rescue the inhibitory effect of schisandrin A during osteoclastogenesis. In addition, inhibition of Nrf2 attenuated the schisandrin A–mediated suppressing of osteoclast transcriptional factor NFATc1 and c‐Fos. However, the underlying mechanisms of how Nrf2 interacts with NFATc1 and c‐Fos in the present study still remain unknown. Taken together, the anti‐oxidant mechanism of schisandrin A on the differentiation of osteoclasts was functioned by Nrf2.

Crosstalk of reactive oxygen species and NF‐κB signalling pathway has been extensively investigated in recent years.^28^ RANKL‐induced reactive oxygen species in BMMs plays an important role during osteogenesis.^5^ Moreover, NF‐κB signalling pathway has been regarded as an pivotal downstream factor in RANKL‐induced differentiation of osteoclast.^29^ In the present study, we observed schisandrin A could inhibit the degradation of IκBα and the phosphorylation of P65, as well as the translocations of P65 into nuclei. Although NF‐κB luciferase has been performed to explore the effect of transcription when BMMs treated with this compound, the underlying mechanisms still needed to be elucidated. Recently, the underlying mechanisms between Nrf2 and NF‐κB signalling pathway in oxidative or inflammatory conditions have been demonstrated in vivo and in vitro.^9^, ^11^, ^12^, ^26^, ^29^, ^30^, ^31^, ^32^ Our study demonstrated that inhibition of Nrf2 increased the phosphorylation of P65 and Iκbα, whereas Sch could hardly rescue the siNrf2‐mediated NF‐κB activation, indicating that Sch suppresses the RANKL‐induced NF‐κB signalling by Nrf2. Nrf2 interacted negatively with NF‐ĸB in inflammation and immune responses.^33^ Rac1 and STING might be involved in this process, one previous study reported that Nrf2‐specific activator inhibits NF‐κB signalling pathway by STING, and hence, Nrf2‐specific activator appeared to be a potential drug in the treatment of osteoporosis by inhibiting NF‐κB.^12^ Some transcriptional factors and proteins also presented as a potential target in inhibiting osteoclasts by suppressing NF‐κB pathway.^30^, ^31^, ^32^ Some compounds also have been reported to be with the effect of anti–osteoclast‐related disease.^11^, ^12^, ^17^, ^34^ However, to which key factor these compounds targeted and functioned still need to be elucidated.

**FIGURE 8:**
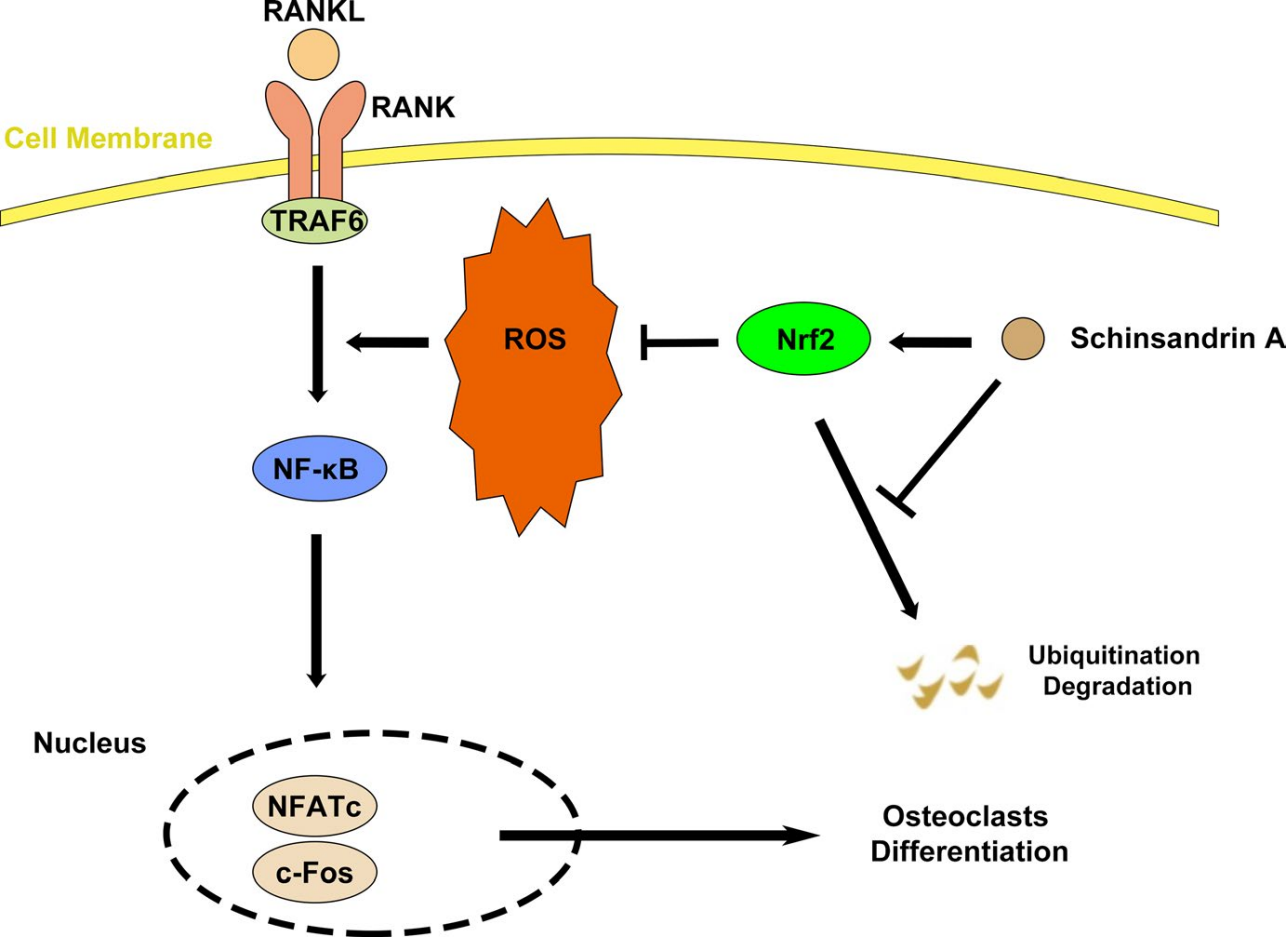
Proposed scheme of Sch restrains osteoclastogenesis by inhibiting reactive oxygen species and activating Nrf2 signalling

In conclusion, the work presented here reveals a new compound schisandrin A could inhibit osteoclastogenesis in vitro and attenuate OVX‐induced osteoporosis in vivo by enhancing the expression of Nrf2 (Figure 8). Thus, screening some specific compounds which enhance the activity or expression of Nrf2 may be a promising alternative in the treatment of osteoclast‐related disease.

## CONFLICT OF INTEREST

The authors have no conflict of interests.

## AUTHOR CONTRIBUTIONS

Shuo Ni and Baoqing Yu designed the study. Zhi Qian, Yin Yuan, Dejian Li and Zeyuan Zhong performed the experiments and drafted the manuscript. Farnaz Ghorbani, Xu Zhang and Fangxue Zhang analysed the data. Zhenhua Zhang and Zichen Liu gave the technical and material supports. All authors read and approved the final manuscript.

## Supporting information

Fig S1

Fig S2

Fig S3

Table S1‐S2

## Data Availability

The data sets used and/or analysed during the current study are available from the corresponding author on reasonable request.
