# Supplementary figures and images for: Schisandrin A restrains osteoclastogenesis by inhibiting reactive oxygen species and activating Nrf2 signalling
Source: Cell Prolif. 2020 Sep 1;53(10):e12882. doi: 10.1111/cpr.12882 (PMC7574870; doi:10.1111/cpr.12882)

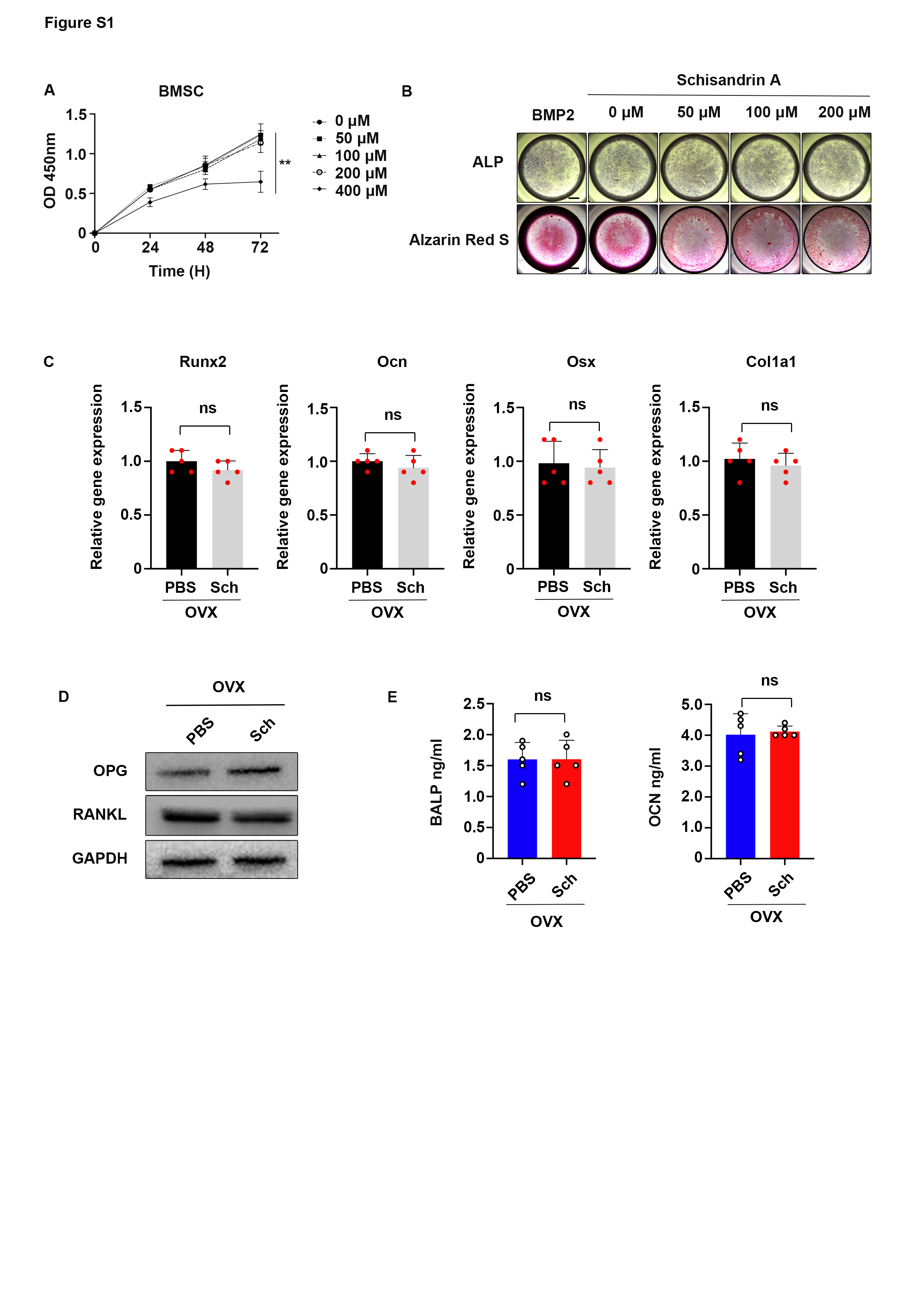

Supplement: Supplementary file 1 — Fig S1 [file CPR-53-e12882-s001.tif]

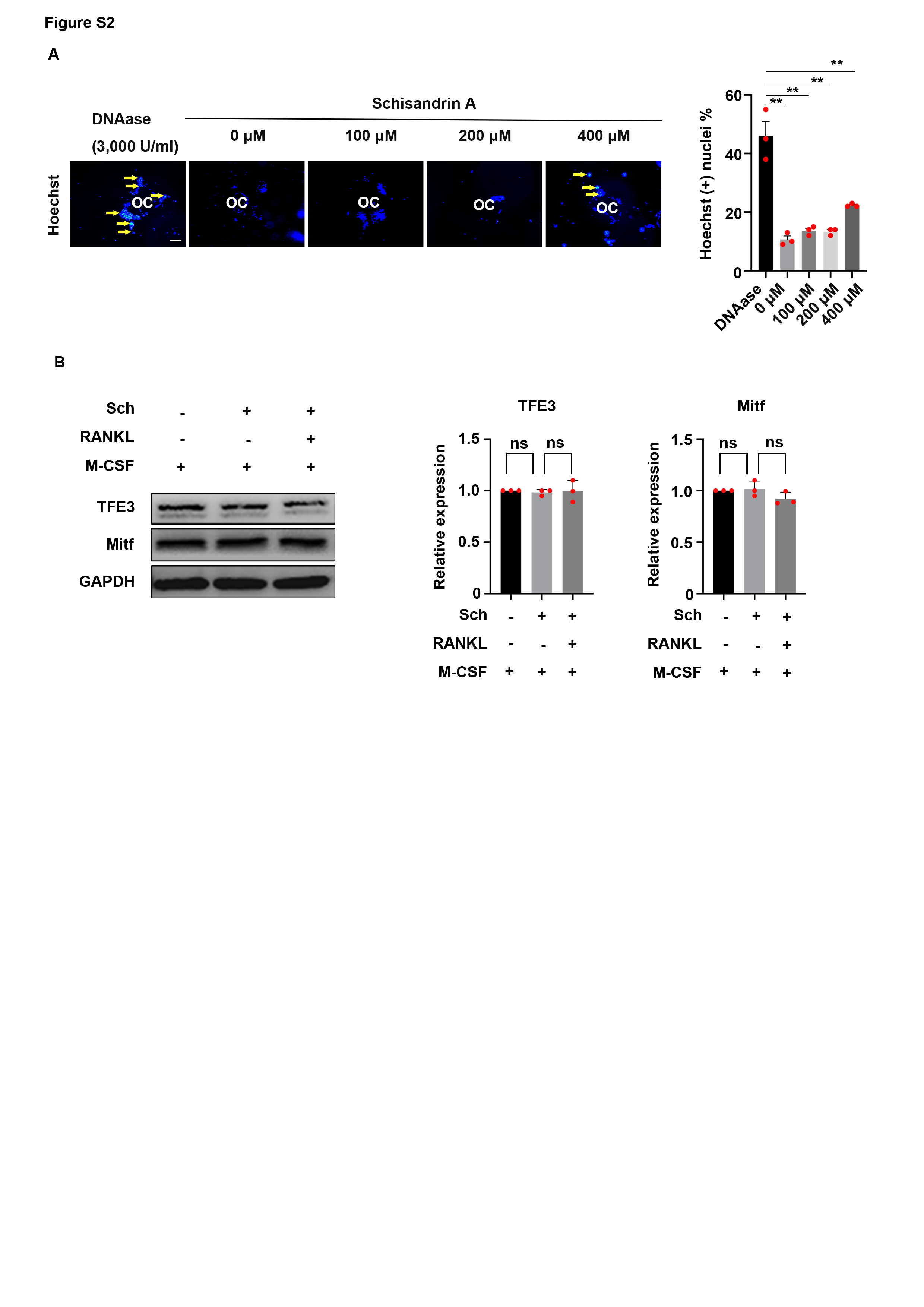

Supplement: Supplementary file 2 — Fig S2 [file CPR-53-e12882-s002.tif]

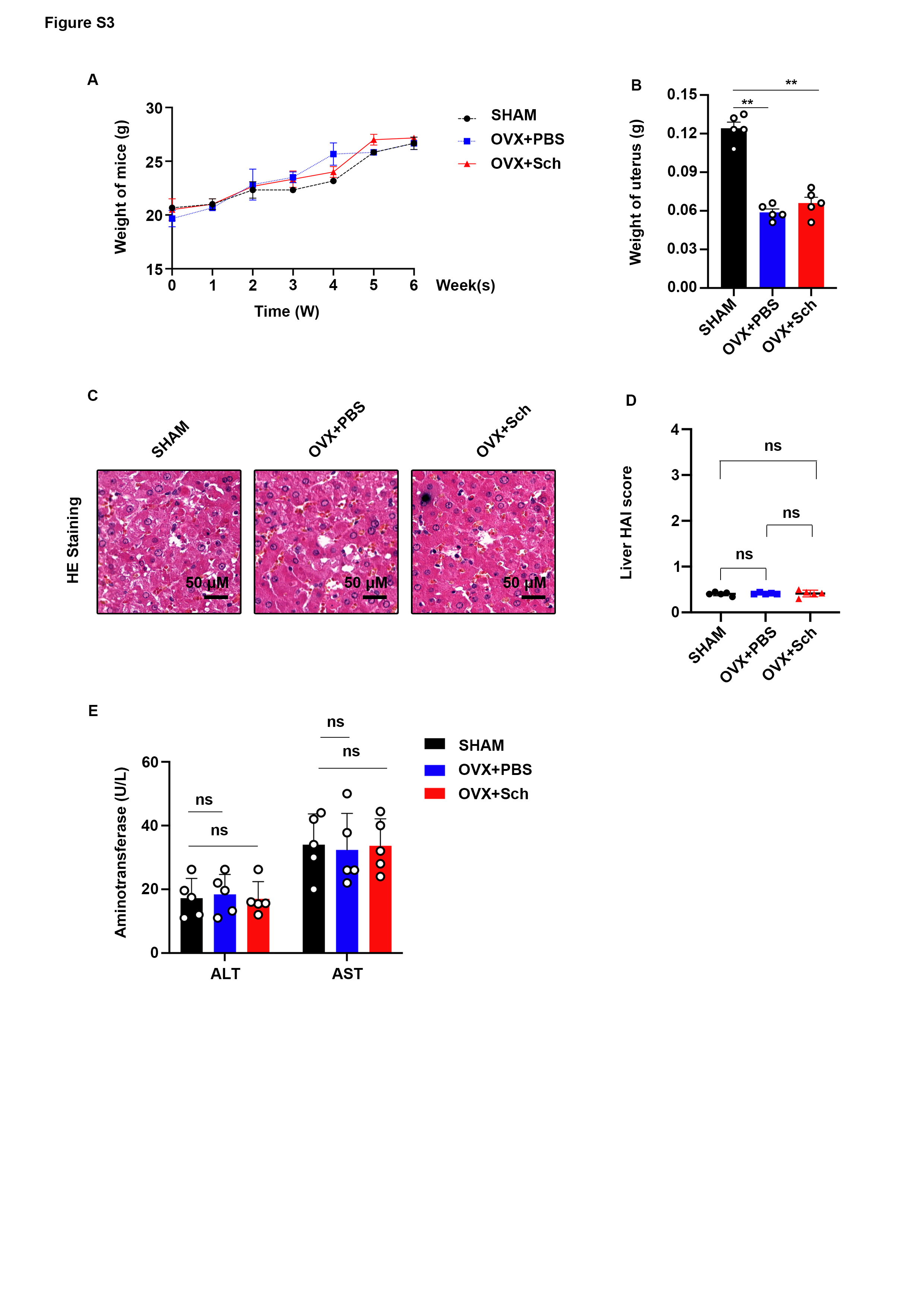

Supplement: Supplementary file 3 — Fig S3 [file CPR-53-e12882-s003.tif]
