# Supplementary material for: Schisandrin A restrains osteoclastogenesis by inhibiting reactive oxygen species and activating Nrf2 signalling
Source: Cell Prolif. 2020 Sep 1;53(10):e12882. doi: 10.1111/cpr.12882 (PMC7574870; doi:10.1111/cpr.12882)
Supplement: Supplementary file 4 — Table S1‐S2 [file CPR-53-e12882-s004.docx]

**Supplemental Table 1. Primers used in qRT-PCR**

| **Genes** | **Forward (5′-3′)** | **Reverse (5′-3′)** |
| --- | --- | --- |
| ***Gapdh*** | ACCCAGAAGACTGTGGATGG | CACATTGGGGGTAGGAACAC |
| ***Nfatc1*** | CAACGCCCTGACCACCGATAG | GGCTGCCTTCCGTCTCATAGT |
| ***c-Fos*** | GCGAGCAACTGAGAAGAC | TTGAAACCCGAGAACATC |
| ***MMP9*** | CGTGTCTGGAGATTCGACTTGA | TTGGAAACTCACACGCCAGA |
| ***TRAcP*** | CACTCCCACCCTGAGATTTGT | CCCCAGAGACATGATGAAGTCA |
| ***Nrf2*** | TCTCCTCGCTGGAAAAAGAA | AATGTGCTGGCTGTGCTTTA |
| ***Runx2*** | TTCTCCAACCCACGAATGCAC | CAGGTACGTGTGGTAGTGAG |
| ***Ocn*** | GAGGGCAATAAGGTAGTGA ACAGA | AAGCCATACTGGTTTGATAGCTCG |
| ***Osx*** | TCGAGGATGGCGTCCTCTCTGC | TGGTGCTTGAGAAGGGAGCTGG |
| ***Col1a1*** | GCTCCTCTTAGGGGCCACT | CCACGTCTCACCATTGGGG |

**Supplementary Table 2. Sequences used in siRNA**

| **Genes** | **Sense sequences** |
| --- | --- |
| **siNrf2** | 5′-UGAAAGCACAGCAGAAUUTT-3′ |
| **siControl** | 5′-GAGCGGCCGAGCAACGUCUAU-3’ |
